# Association between age factors and strategies for promoting participation in gastric and colorectal cancer screenings

**DOI:** 10.1186/s12885-018-4244-6

**Published:** 2018-03-27

**Authors:** Chisato Hamashima, Hiroshi Sano

**Affiliations:** 1 Division of Cancer Screening Assessment and Management, Center for Public Health Science, National Cancer Center, 5-1-1 Tsukiji Chuo-ku, Tokyo, 104-0045 Japan; 2 Faculty of Economics, Shiga University, 1-1-1 Baba-cho, Hikone, Japan

**Keywords:** Gastric cancer screening, Colorectal cancer screening, Participation rate, Invitation letter, Target age group, Older people

## Abstract

**Background:**

Despite the long history of cancer screening in Japan, the participation rates in gastric and colorectal cancer screenings have not increased. Strategies for improving the participation rates have been proposed, but differences in their effects among different age groups remain unclear.

**Methods:**

The Japanese government conducted a national survey in all municipalities in Japan in 2010 to investigate whether the implementation of promotion strategies increased participation in cancer screening. We investigated the association between age factors and strategies for promoting participation in cancer screening based on this national survey. Multiple regression analysis with generalized linear model was performed using the participation rates in gastric and colorectal cancer screenings as dependent variables, and the following strategies for promoting participation as independent variables: 1) personal invitation letters, 2) household invitation letters, 3) home visits by community nurses, 4) screenings in medical offices, and 5) free cancer screening programs.

**Results:**

One thousand six hundred thirty nine municipalities for gastric cancer screening and 1666 municipalities for colorectal cancer screening were selected for the analysis. In gastric and colorectal cancer screenings, the participation rates of individuals aged 60–69 years was higher than those of other age groups. Personal and household invitation letters were effective promotion strategies for all age groups, which encouraged even older people to participate in gastric and colorectal cancer screenings. Screening in medical offices and free screenings were not effective in all age groups. Home visits were effective, but their adoption was limited to small municipalities.

**Conclusions:**

To clarify whether promotion strategies can increase the participation rate in cancer screening among different age groups, 5 strategies were assessed on the basis of a national survey. Although personal and household invitation letters were effective strategies for promoting participation in cancer screening for all age groups, these strategies equally encouraged older people to participate in gastric and colorectal cancer screenings. If resource for sending invitation letters are limited, priority should be given to individuals who are in their 50s and 60s for gastric and colorectal cancer screening.

## Background

Cancer screening is one of the major strategies for controlling cancer worldwide. To achieve the goal of reducing mortality from the target cancer, a high participation rate is required as well as quality assurance of evidence-based screening. Gastric cancer screening as a national program in Asia has been carried out only in Korea and Japan [1, 2]. However, colorectal cancer screening by fecal occult blood testing (FOBT) has been gradually adopted worldwide [1–3]. Although colorectal cancer screening has not yet been fully established compared with breast and cervical cancer screenings, some countries have already introduced organized screening which clearly define the target age group and encourage them to participate using a call/recall system [1, 3, 4]. Usually, the upper age limit in colorectal cancer screening ranges from 64 to 80 years [3].However, there is no upper age limit in the national programs for gastric and colorectal cancer screenings in Korea and Japan [1, 5].

Although there is a long history of cancer screening in Japan, there is unfortunately still no national call/recall system [5]. Therefore, the participation rates have gradually decreased or flattened in all screening programs. In fact, the national average participation rates have not increased, particularly for gastric cancer screening at 10.1% and for colorectal cancer screening at 21.1% in 2012 [6]. Therefore, in 2009, the Japanese government published cancer control plans and revised them in 2012, which paved the way for setting a national goal for reducing cancer mortality by improvement of cancer prevention, screening, and treatment [7]. In these plans, the goal was to achieve a participation rate of 40% for gastric and colorectal cancer screenings within 5 years, and the national government has actively encouraged improving participation rates in cancer screening [7]. Then, municipal governments have uniquely undertaken strategies for increasing the participation rate in cancer screening in accordance with their own responsibilities.

Although the incidences of gastric and colorectal cancers increase with age, individuals who are in their 50s and 60s have priory as the target populations for gastric and colorectal cancer screenings based on their incidence and productivity loss. As there is no upper age limit in Japan [5], promotion strategies for cancer screening have equally targeted all age groups. Thus, individuals aged 70 years and older can easily participate in cancer screening, as evidenced by the recent increase in their participation rate [6]. In a previous study in Japan, invitation letter and personal visits by community nurses were shown to increase participation in cancer screening [8]. However, the different effects of various promotion strategies on individuals aged 70 years older and younger remain unclear. In this article, we investigated the association between age factors and strategies for promoting participation in cancer screening based on a national survey.

## Methods

### Cancer screening programs in Japan

In Japan, the national government has defined the national policy of cancer screening programs based on the law and has selected screening methods as per evidence-based guidelines [2]. Five cancer screening programs including gastric and colorectal cancer screenings are provided by local municipal governments as financially supported by the national government. Cancer screening programs are not covered by health insurance, but are supported by tax. Gastric cancer screening using the upper gastrointestinal series with barium meal (UGI) was introduced as a national program in 1983, followed by colorectal cancer screening using FOBT in 1992 [2]. The target age groups of gastric and colorectal cancer screenings are individuals aged 40 years and older and the screening interval is every year. Most programs have been mainly provided in the form of mass screening by mobile vans and in public places including local cancer screening centers. Opportunities for cancer screening in medical offices have been mainly provided in urban areas.

### Data sources

For this analysis, we used the results of 2 national surveys for all municipalities. The first is annul survey of cancer screening programs [9]. The second is a specific survey regarding the promotion plan for cancer screening in 2010 [10].

The first data source is annual reports that all municipalities submit to the national government showing the results of their cancer screening programs. The results include the total numbers of participants, positive cases of primary screening, examinees of the diagnostic examinations, and cancer detection rates. Although the total number of municipalities in 2010 was 1746, 46 municipalities failed to submit the results of their cancer screening because of the Great East Japan Earthquake [9].

Based on the cancer control plan by the national government, local municipalities have individually undertaken promotion strategies to improve participation rates in their cancer screenings. In 2010, a national survey was temporally conducted for all municipalities to investigate the effect of ‘with or without the implementation’ of the following promotion strategies for increasing participation in cancer screening: 1) personal invitation letters, 2) household invitation letters, 3) home visits by community nurses, 4) screenings in medical offices, and 5) free cancer screening programs [11]. These promotion strategies have often been introduced in local municipalities. Some municipalities send invitation letters directly to target individuals, or other municipalities send them to family units regardless of the total number of target individuals. Although cancer screening has been mainly provided as mass screening in Japan, individual participation in cancer screening at medical offices can increase access. These also correspond to the basic concept of the standard promotion strategies which are assessed by the Community Preventive Service Task Force [11] as follows: personal and household invitations as client reminder, home visits by community nurses as one-on-one education, screenings in medical offices to reduce structure barrier, and free cancer screening programs to reduce out-of-pocket payments.

To refine the target municipalities for our analysis based on above-mentioned data source, the following municipalities were excluded: 1) no promotion strategies for improving the participation rate in the second survey, 2) missing or inconsistent data related to the number of participants in the first survey, 3) less than 100 participants in gastric and colorectal cancer screenings in 2010.

### Statistical analysis

To investigate the associations and strategies for improving the participation rates in all cancer screenings, we defined the calculation method of the participation rate as follows: the denominator was defined as the National Census population in 2010 [12], and the numerator was defined as the number of participants in each cancer screening cited from the report on regional public health services and health promotion in 2010 [9]. The participation rates in gastric and colorectal cancer screenings were calculated by 4 age groups as follows: 40–49 years, 50–59 years, 60–69 years, and 70 years or older.

Multiple regression analysis with generalized linear model (GLM) was performed using the participation rates in gastric and colorectal cancer screenings as dependent variables, and the strategies for promoting participation as independent variables. As the participation rate ranged from 0 to 1, binominal distribution was assumed for dependent variables. Although the participation rate was a continuous variable, ordinary least squares (OLS) assuming a normal distribution as an error term could not be used for regression analysis. Factors that affected participation rates were estimated by GLM using logit function as link function. Marginal effects, similarly to the coefficient value in OLS, represents a change in the explained variable due to one unit change in the explanatory variable in GLM. In GLM, when the marginal effect of a certain strategy is estimated as β, it means that the participation rate is increased 12 by (β × 100) % points.

For all the promoting strategies consisting of personal invitation letters, household invitation letters, home visits by community nurses, screenings in medical offices, and free cancer screenings for all age groups, a dichotomous variable that takes the value 0 or 1 was used to indicate whether they are implemented. The total population of the municipalities was divided into 2 groups, namely, ≥ 30,000 and < 30,000 because the city and town were divided by the total population as 30,000. The total number of medical facilities was considered as an important factor affecting cancer screening in medical facilities, which were cited from the Medical Institute Survey in 2010 [13]. The total population (≥ 30,000 or < 30,000), the total number of medical facilities per 1000 individuals aged 40 years and older, and the proportion of women were used as covariates. The estimates of all strategies were adjusted by these covariates.

Statistical analysis was performed using STATA 13.0 (STATA, College Station, TX, USA). All test statistics were two-tailed, and *P*-values < 0.05 were considered to indicate a statistically significant difference.

## Results

**Fig. 1:**
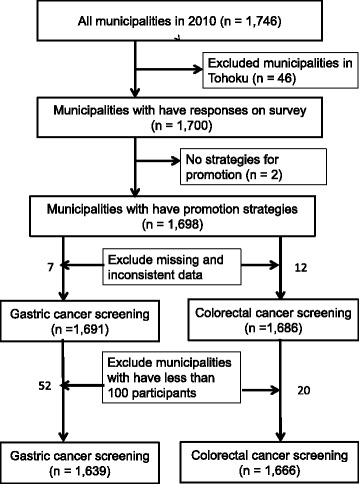
Flowchart of the selection process for the target group. Although the total number of municipalities in 2010 was 1746, 46 municipalities failed to submit the results of their cancer screening because of the Great East Japan Earthquake [8]. Two municipalities were not able to conduct the above-mentioned questionnaire survey on promotion strategies for improving the participation rate. Municipalities that had missing or inconsistent data related to the number of participants were excluded as follows: 7 municipalities for gastric cancer screening and 12 municipalities for colorectal cancer screening. In addition, there were municipalities whose number of participants was less than 100, namely, 52 municipalities for gastric cancer screening and 20 municipalities for colorectal cancer screening. These municipalities were also excluded from the analysis [8]. Finally, 1639 municipalities for gastric cancer screening and 1666 municipalities for colorectal cancer screening were analyzed

The selection procedure for the target population is shown in Fig. 1. From 1746 municipalities in 2010, 1639 municipalities for gastric cancer screening and 1666 municipalities for colorectal cancer screening were analyzed.

In gastric cancer screening, the participation rate of individuals aged 60–69 years was higher than the participation rate of individuals of other age groups. The female ratios were almost the same for all age groups, but statistically significant differences were shown owing to the slightly lower rates for the 60–69 age group (Table 1). The most common promotion strategy was screening at medical facilities, with 45% of the municipalities preparing the screening opportunities. Home visits by community nurses were limited to 6%.

**Table 1:** Basic characteristics of gastric cancer screening

	Average	95%CI		*P*-value
Total number of municipalities	1639			
Participation rate (%)				
40–49 years	13.18	12.60	13.77	< 0.05
50–59 years	14.90	14.29	15.50	
60–69 years	21.58	20.89	22.26	
≥ 70 years	14.06	13.48	14.64	
Intervention to promote participation in cancer screening				
Personal invitation letters	0.384	0.360	0.407	< 0.05
Household invitation letters	0.214	0.194	0.234	
Home visits by community nurses	0.059	0.047	0.070	
Screenings at medical offices	0.448	0.424	0.473	
Free screenings for all age groups	0.081	0.068	0.094	
Covariate				
≥ 30,000 of total population in each municipality	0.475	0.450	0.499	–
Total number of medical facilities per 1000 individuals aged 40 years and older	1.165	1.133	1.197	–
Percentage of women (%)				
40–49 years	59.59	59.05	60.14	< 0.05
50–59 years	59.82	59.27	60.38	
60–69 years	56.01	55.74	56.28	
≥ 70 years	60.13	59.92	60.34	

Statistically significant difference based on ANOVA (analysis of variance)

In colorectal cancer screening, the participation rate in the group consisting of individuals who were in their 60s and 70s was higher than the participation rate in the age group consisting of individuals who were in their 40s and 50s (Table 2). The most common promotion strategy was screening at medical offices, with 50% of the municipalities preparing the screening opportunities. Sending personal invitation letters was performed in 38% of the municipalities for gastric and colorectal cancer screenings.

**Table 2:** Basic characteristics of colorectal cancer screening

	Average	95%CI		*P* value
Total number of municipalities	1666			
Participation rate (%)				
40–49 years	15.69	15.04	16.33	< 0.05
50–59 years	18.84	18.17	19.52	
60–69 years	28.70	27.95	29.46	
≥ 70 years	21.89	21.17	22.61	
Intervention to promote participation in cancer screening				
Personal invitation letters	0.381	0.357	0.404	< 0.05
Household invitation letters	0.221	0.202	0.241	
Home visits by community nurses	0.061	0.049	0.072	
Screenings at medical offices	0.502	0.478	0.526	
Free screenings for all age groups	0.092	0.079	0.106	
Covariate				
≥ 30,000 of total population in each municipality	0.468	0.444	0.492	–
Total number of medical facilities per 1000 individuals aged 40 years and older	1.183	1.147	1.219	–
Percentage of women (%)				
40–49 years	59.31	58.77	59.85	< 0.05
50–59 years	59.69	59.14	60.25	
60–69 years	56.09	55.83	56.35	
≥ 70 years	60.35	60.15	60.55	

Statistically significant difference based on ANOVA (analysis of variance)

The results of the multiple regression analysis for gastric cancer screening are shown in Table 3. Home visits by community nurses were identified as the most effective promotion strategy in all age groups. Sending personal and household invitation letters were also effective in all age groups. Although sending household invitation letters was more effective in the age group consisting of individuals who were in their 40s to 60s, the marginal effects were similar in sending personal invitation letters and home visits by community nurses in all age groups. However, the marginal effects were limited to below 10% point. Negative effects of screening at medical offices were observed in the age group consisting of individuals who were in their 40s to 60s, but these were not significant. The marginal effects of free screenings were not significant in all age groups.

**Table 3:** Effective factors for promoting participation in gastric cancer screening

	40–49 years				50–59 years				60–69 years				≥ 70 years			
	Marginal effect	95%CI		*P*-value	Marginal effect	95%CI		*P*-value	Marginal effect	95%CI		*P*-value	Marginal effect	95%CI		*P*-value
Personal invitation letters	**0.050**	**0.017**	**0.084**	0.003	**0.053**	**0.018**	**0.089**	0.003	**0.057**	**0.017**	**0.098**	0.006	**0.045**	**0.011**	**0.080**	0.010
Household invitation letters	**0.056**	**0.018**	**0.095**	0.004	**0.060**	**0.020**	**0.100**	0.004	**0.078**	**0.031**	**0.124**	0.001	**0.044**	**0.004**	**0.084**	0.030
Home visits by community nurses	**0.079**	**0.019**	**0.138**	0.010	**0.091**	**0.029**	**0.153**	0.004	**0.097**	**0.021**	**0.172**	0.012	**0.084**	**0.024**	**0.144**	0.006
Screenings at medical offices	−0.012	−0.045	0.021	0.484	−0.015	−0.050	0.020	0.401	−0.010	− 0.050	0.030	0.614	0.000	−0.034	0.034	0.998
Free screenings for all age groups	0.050	−0.005	0.105	0.073	0.037	−0.022	0.097	0.222	0.027	−0.044	0.098	0.456	0.006	−0.054	0.066	0.842
Total number of municipalities	1639				1639				1639				1639			
Log likelihood	− 457.97				− 490.82				− 595.76				− 472.63			
AIC	0.5698				0.6099				0.738				0.5877			

Adjusted by total population (≥ 30,000 or < 30,000), total number of medical facilities per 1000 individuals aged 40 years and older, and proportion of women

Boldface indicates statistical significance at *P* < 0.05

The results of the multiple regression analysis for colorectal cancer screening are shown in Table 4. Sending personal and household invitation letters and visits by community nurses were also effective in all age groups. However, the marginal effects of sending personal and household invitation letters were higher in individuals aged 70 years and older than in individuals aged 40–69 years. The marginal effects of screening at medical offices and free screenings were not significant in all age groups.

**Table 4:** Effective factors for promoting participation in colorectal cancer screening

	40–49 years				50–59 years				60–69 years				≥ 70 years			
	Marginal effect	95%CI		*P*-value	Marginal effect	95%CI		*P*-value	Marginal effect	95%CI		*P*-value	Marginal effect	95%CI		*P*-value
Personal invitation letters	**0.055**	**0.020**	**0.091**	0.002	**0.061**	**0.023**	**0.099**	0.002	**0.065**	**0.021**	**0.110**	0.004	**0.056**	**0.015**	**0.096**	0.007
Household invitation letters	**0.063**	**0.023**	**0.103**	0.002	**0.070**	**0.026**	**0.113**	0.002	**0.088**	**0.037**	**0.139**	0.001	**0.055**	**0.007**	**0.102**	0.023
Home visits by community nurses	**0.088**	**0.025**	**0.151**	0.006	**0.101**	**0.033**	**0.169**	0.004	**0.103**	**0.018**	**0.187**	0.017	**0.099**	**0.024**	**0.175**	0.010
Screenings at medical offices	−0.021	−0.057	0.014	0.234	−0.023	−0.061	0.015	0.228	−0.010	−0.055	0.034	0.643	0.025	−0.016	0.065	0.230
Free screenings for all age groups	0.036	−0.022	0.094	0.219	0.039	−0.024	0.101	0.224	0.040	−0.033	0.113	0.280	0.035	−0.029	0.100	0.281
Total number of municipalities	1666				1666				1666				1666			
Log likelihood	− 512.74				− 566.53				− 688.6				− 617.9			
AIC	0.6263				0.6909				0.8375				0.7526			

Adjusted by total population (≥ 30,000 or < 30,000), total number of medical facilities per 1000 individuals aged 40 years and older, and proportion of women

Boldface indicates statistical significance at *P* < 0.05

## Discussion

Although we identified that personal and household invitation letters were effective promotion strategies for increasing participation in gastric and colorectal cancer screenings [8], we obtained the same results even if the subjects were divided into 4 age groups. These results are also consistent with those of previous studies [4, 14, 15]. Sending invitation letters to the target population is a central role of the call/recall system for cancer screening programs. The results suggest that the call/recall system could be adopted to improve the participation rate in Japan. Although home visits also encouraged participation in cancer screening, a limited number of municipalities introduced this strategy mainly in rural areas whose total population was < 30,000. Since face-to-face communication is performed by community nurses, the same effects as those of a physician’s recommendation can be expected.

Personal and household invitation letters had similar effects for all age groups. Client reminder was sufficient to increase the participation rate for all target populations in colorectal cancer screening [14]. However, there is still no clear upper age limit and older people can continue to participate in cancer screening. Among older veterans, it has been reported that 41% of the patients with severe comorbidity and life expectancies of less than 5 years were screened for colorectal cancer [16]. Lewis et al. also reported that older participants would continue cancer screening throughout their lives, and 43% would consider cancer screening even if their doctor does not recommend it [17]. Some older people have continued cancer screening as a matter of routine or habit, or occasionally in response to a physician’s prompting and an invitation letter [18]. In gastric and colorectal cancer screenings, the main participants were individuals aged 60–69 years, and they continued to be screened even after 70 years. As a result, invitation letters encouraged older people to participate in cancer screening. If life expectancy is short, the net benefit is limited for older people [19]. Despite the high possibility of harms in the forms of overdiagnosis and complications for older people, they might still hold unrealistic expectations. As most brochures related to cancer screening tend to show favorable results of participation in cancer screening [20, 21], older people cannot easily distinguish and separate the necessity and effects of cancer screening. However, in spite of their frequent visits to the medical offices, older people who continue cancer screening still have few opportunities to be informed of the potential benefits and harms of cancer screening [18]. Informed decision making has been assumed to resolve these issues, but this has not been effective as expected [17, 18, 22].

Colorectal cancer screening has been increasingly performed following breast and cervical cancer screenings; however, some countries have not yet introduced such screening or have remained in the pilot phase [3]. The coverage of invitation in the target age population is lower in colorectal cancer screening than in breast and cervical cancer screenings. The major reason for this lower coverage was resource limitations, particularly for total colonoscopy for diagnostic examination. Although the European Union defined the target population as 50–74 years, some countries (e.g., UK) intensively targeted a narrow range age group [3]. On the other hand, there is no upper age limit in the Czech Republic, Korea, and Japan [1–3]. For example in breast cancer screening, individuals who are in their 60s have continued participating even more and have maintained a higher participation rate than individuals who are in their 50s [23]. If the target age is not clearly defined, it is more favorable for the older age group to continue participating in cancer screening. Since an invitation letter has a similar effect on individuals who are in their 70s, the priority of sending invitation letters targeting individuals who are in their 50s and 60s can be a solution for the efficient use of limited resources for gastric and colorectal cancer screenings.

There are several limitations in this study. First, although the response rates were high, several municipalities could not provide sufficient response or reply for analysis. Since the participation rates were higher in the Tohoku areas, their responses might have been affected. Additional analysis is needed after the recovery from the Great East Japan Earthquake, although some municipalities have not yet completely recovered. Second, our study is based on a provider perspective and not on individuals. However, individual participation is affected by various factors including cultural background. To improve the participation rate in cancer screening, these factors should be considered. Third, the subjects who were sent invitation letters were different among the municipalities. Some municipalities have already introduced a similar system, but such system might be different from the original call/recall system for sending invitation to all individuals of the target population for the cancer screening programs. Municipal governments often choose a limited population who will benefit from the local government health insurance and not those who have employment insurance. Therefore, all people cannot receive the same benefits of encouragement from invitation letters even if they have no opportunities to have cancer screening in their workplace. Finally, the Japanese health insurance system also influences participation in cancer screening. Although the Japanese health insurance does not cover cancer screening, all citizens can easily access medical services, with most of these serves covered by small co-payments [24]. Therefore, people do not feel obligated to avail of the municipal cancer screening programs. However, promotion strategies may become more effective under a different healthcare system. In Korea, the call/recall system is a major reason for the increase in the participation rate of cancer screening [15]. Therefore, it may be possible to generalize the results to other countries.

## Conclusion

To clarify promotion strategies for increasing the participation rate in cancer screening among different age groups, 5 strategies were assessed on the basis of a national survey. Home visits were effective as a strategy, but their adoption was limited to small municipalities. Screening at medical offices and free screening were not effective in all age groups. Although personal and household invitation letters were effective strategies for promoting participation in cancer screening for all age groups, these strategies equally encouraged older people to participate in gastric and colorectal cancer screenings. If resources are limited particularly in sending invitation letters, priority should be given to individuals who are in their 50s and 60s for gastric and colorectal cancer screenings.
